# Prevalence of Liver Fibrosis and its Association with Non-invasive Fibrosis and Metabolic Markers in Morbidly Obese Patients with Vitamin D Deficiency

**DOI:** 10.1007/s11695-016-2123-2

**Published:** 2016-03-17

**Authors:** Maria Luger, Renate Kruschitz, Christian Kienbacher, Stefan Traussnigg, Felix B. Langer, Karin Schindler, Tanja Würger, Friedrich Wrba, Michael Trauner, Gerhard Prager, Bernhard Ludvik

**Affiliations:** 1 Division of Endocrinology and Metabolism, Department of Internal Medicine III, Medical University of Vienna, Vienna, Austria; 2 Special Institute for Preventive Cardiology And Nutrition – SIPCAN save your life, Salzburg, Austria; 3 Division of Gastroenterology and Hepatology, Department of Internal Medicine III, Medical University of Vienna, Vienna, Austria; 4 Division of General Surgery, Department of Surgery, Medical University of Vienna, Vienna, Austria; 5 Department of Pathology, Medical University of Vienna, Vienna, Austria; 6 1st Department of Medicine and Karl Landsteiner Institute for Obesity and Metabolic Diseases, Rudolfstiftung Hospital, Vienna, Austria

**Keywords:** Liver fibrosis, Metabolic syndrome, Diabetes mellitus, Vitamin D, Bariatric surgery

## Abstract

**Background:**

Morbidly obese patients are at risk for non-alcoholic fatty liver disease (NAFLD) and vitamin D deficiency (VDD). Non-alcoholic steatohepatitis (NASH) is the progressive variant of NAFLD and can advance to fibrosis, cirrhosis, and liver cancer. We aimed to examine prevalence of liver fibrosis and its non-invasive predictors in bariatric patients with VDD (<75 nmol/l).

**Methods:**

Baseline liver biopsy of a randomized controlled trial was performed in 46 patients with omega loop gastric bypass. Clinical, laboratory, and histological data were examined and tested with univariate and multivariable analysis.

**Results:**

In total, 80 % were females, aged 42 (SD 13) years with BMI 44 (4) kg/m^2^. Twenty-six percent had diabetes mellitus (DM) and 44 % metabolic syndrome (MeS). Seventy-two percent had NASH, 11 % simple steatosis, and 17 % normal liver. In total, 30 % demonstrated significant fibrosis (*F* ≥ 2) with 9 % of advanced (F3) and 4 % cirrhosis (F4). Increased stages of fibrosis were primarily associated with higher levels of HOMA2-insulin resistance (IR), procollagen type I propeptide (P1NP), lower osteocalcin, albumin-corrected calcium, parathyroid hormone, vitamin D, male sex, and higher age. Other independent risk factors for advanced fibrosis were MeS (OR = 9.3 [0.99–87.5], *p* = 0.052) and DM (OR = 12.8 [1.2–137.4], *p* = 0.035). The fibrosis FIB-4 index <10.62 and NAFLD fibrosis score <−26.93 had a negative predictive value of 100 and 96 %, respectively.

**Conclusions:**

Liver fibrosis is frequent in morbidly obese patients with concurrent DM and/or MeS. Increased serum levels of IR, P1NP, lower osteocalcin, and VDD are clinically relevant predictors of fibrosis. Consequently, we suggest that patients with preoperative presence of these markers are at increased risk for liver fibrosis and should be monitored closely.

## Introduction/Purpose

There is growing evidence that obesity, diabetes mellitus type 2 (DM), and the metabolic syndrome (MeS) are associated with non-alcoholic fatty liver disease (NAFLD) [1]. NAFLD consists of a wide spectrum ranging from relatively benign hepatic steatosis to more severe non-alcoholic steatohepatitis (NASH) with different stages of fibrosis ultimately progressing to cirrhosis and hepatocellular cancer [2]. Moreover, the association between diabetes and advanced liver disease may have important clinical implications, particularly in morbidly obese patients, and insulin resistance might play an important role for fibrogenesis and carcinogenesis in the liver [3]. In the Verona Diabetes Study, the authors found that liver cirrhosis was the fourth leading cause of death [4]. Furthermore, in bariatric patients, steatosis has been reported in 85–98 %, NASH in 24–98 %, advanced fibrosis (*F* ≥ 3) in 4–16 %, and cirrhosis in 1–7 % [5]. Moreover, low vitamin D levels, which have been associated with insulin resistance (IR) and fibrogenesis [6], might also contribute to the development of NAFLD [7]. However, the mechanisms underlying the association of vitamin D and NAFLD are not yet fully understood and it remains unclear whether other vitamin D-related parameters, e.g., calcium (Ca), 25-hydroxy vitamin D_3_ (25(OH)D_3_), activated 1α,25-dihydroxy vitamin D_3_ (1α,25(OH)_2_D_3_), or parathyroid hormone (PTH) also have a predictive value for disease progression. Of note, progressive liver disease itself may lead to impaired vitamin D metabolism [8, 9]. Moreover, morbidly obese patients with vitamin D deficiency (VDD) may be at increased risk for liver-related mortality [10].

Therefore, the purpose of this analysis was to assess the prevalence of liver fibrosis, to explore the role of VDD and other additional metabolic markers as risk factors for higher stages of liver fibrosis, and the potential of these variables to discriminate for advanced fibrosis (*F* ≥ 3) in morbidly obese patients undergoing bariatric surgery.

## Materials and Methods

From April 2014 to April 2015, consecutive patients, older than 18 years, planned to undergo omega loop gastric bypass, were recruited. The data for the present analysis are the result of baseline measurements from subjects who participated in the LOAD-study, a prospective, double-blind, randomized-controlled vitamin D supplementation trial [11]. Alcohol intake was evaluated during preoperative dietary counseling on basis of the nutritional protocols and was less than 20 or 10 g alcohol per day in men and women. Other causes of liver disease (i.e., viral hepatitis) were excluded by liver histology.

Informed consent was obtained from each patient included in the study. The trial was approved by the local Ethical Committee of the Medical University of Vienna (Ref No.: 1899/2013) and the Austrian Competent Authority (Ref. No.: LCM-718280-0001) and conforms to the ethical guidelines of the 1975 Declaration of Helsinki [12]. Furthermore, the protocol was registered at clinicaltrials.gov (NCT02092376) and the European Clinical Trials Database (EudraCT: 2013-003546-16). The study methods are in accordance with the Consolidated Standards of Reporting Trials (CONSORT) guidelines for reporting randomized trials [13].

### Clinical and Laboratory Assessment

Age, sex, medical history (e.g., comorbidities), and anthropometric data [weight, height, and waist circumference (WC)] were collected [11]. After a 12-h overnight fast, venous blood samples of each participant were obtained to determine the liver enzymes [aspartate aminotransferase (ASAT), alanine aminotransferase (ALAT), γ-glutamyl transpeptidase (GGT)] [11]. Measured serum calcium concentration was corrected for serum albumin [14]. Deficiency and insufficiency for 25(OH)D_3_ was defined with <50 nmol/l and 50–74.9 nmol/l, respectively, [15] and sufficiency with >75 nmol/l. The glomerular filtration rates (GFR) were calculated [16]. Moreover, we used the updated homeostatic model assessment (HOMA2) which calculates insulin resistance (IR) [17]. Metabolic syndrome was defined according to the International Diabetes Federation (IDF) global consensus definition [18]. Additionally, we calculated non-invasive fibrosis marker: the fibrosis FIB-4 index and NAFLD fibrosis score (NFS) [19, 20].

### Liver Biopsy and Histopathological Evaluation

Fine needle trucut biopsies were performed during the laparoscopic omega loop gastric bypass surgery. All tissues were fixed in 10 % buffered formalin and embedded in paraffin. After processing, three histochemical stains (Hematoxylin and Eosin, Chromotrope Aniline Blue, and Prussian blue iron stains) were analyzed and interpreted by two experienced board-certified pathologists, who were unaware of the clinical data (FW, TW). The histological scoring system NAFLD activity score (NAS; from 0 to 8) by *Kleiner* et al. [21] was used to evaluate the grade of steatosis (0–3), hepatocyte ballooning (0–2), lobular inflammation (0–3), and stage of fibrosis with a four-point scale. For histological diagnosis of definite NASH, the diagnostic algorithm by *Bedossa* et al. was used as this algorithm segregates lesions into normal liver, simple steatosis, or NASH and was built based on semiquantitative evaluation of steatosis, hepatocellular ballooning, and lobular inflammation in morbidly obese patients [22].

### Statistical Analysis

Data are presented as mean (standard deviation) for continuous variables and as percentages for categorical variables. Proofing of normal distribution a visual inspection was used and the Kolmogorov–Smirnov test in addition. Independent sample *t* tests, the Mann–Whitney U test or Chi^2^ tests were performed to compare groups. Moreover, the associations of various factors (e.g., steatosis, fibrosis) were assessed by multiple linear regression models with backward selection of variables at a *p*-value threshold of 0.20. All 67 variables (characteristics and laboratory parameters) were tested for significance in a simple linear regression and if significant these variables were entered in the regression model. In addition, a binary logistic regression analysis was performed to identify independent variables associated with advanced fibrosis (*F* ≥ 3). Adjustment for age, sex, and BMI, if appropriate, was used to correct for effect modifiers. The statistical assumptions for regression analyses were met in each case. Moreover, the accuracy of calculated non-invasive algorithms for detection of advanced fibrosis was assessed using receiver operator characteristic (ROC) curves described as area under the curve (AUC) with standard errors, the negative (NPV) and positive predictive value (PPV). Means were compared unadjusted without imputation of missing data. The significance level was set at *p* ≤ 0.05. The analyses were performed with the SPSS 23.0 (IBM Corporation, NY, US)

## Results

### Characteristics of the Study Population

A total of 135 morbidly obese patients were recruited and screened for study inclusion and exclusion criteria. Eighty-five patients were excluded (68 not meeting criteria, 17 declined to participate). Fifty patients were included in the study. Out of these, 46 underwent a liver biopsy during the laparoscopic omega loop gastric bypass surgery (4 liver biopsies were omitted due to logistical reasons).

Caucasian was the predominant ethnicity (90 %) followed by African (4 %), Latin American (2 %), and other (2 %). Twenty-six percent were diagnosed with DM, 52 % hypertension, and 48 % hypertriglyceridemia. Moreover, 44 % met the criteria for MeS and an additional 30 % exhibited at least one to two components of the MeS. Table 1 shows all parameters and liver histology according to the metabolic syndrome as well as characteristics of the study population.

**Table 1:** Parameters (selected) and liver histology according to the metabolic syndrome

	Total (*n* = 50)	No MeS (*n* = 28)	MeS (*n* = 22)	*p* value
Age (years)	42 (13)	36 (13)	51 (7)	*< 0.001*
Sum drugs (*n*)	6 (8)	2 (3)	10 (10)	*< 0.001*
Weight (kg)	120.1 (13.3)	122.0 (13.1)	117.6 (13.3)	0.247
BMI (kg m^−2^)	43.8 (4.3)	44.5 (4.8)	42.9 (3.5)	0.190
WC (cm)	127.4 (10.6)	127.7 (11.0)	127.0 (10.3)	0.802
Corr. Ca (mmol/l)	2.2 (0.1)	2.2 (0.1)	2.2 (0.1)	0.502
PTH (pg/ml)	48.7 (14.3)	52.2 (13.9)	44.3 (14.0)	0.054
25(OH)D_3_ (nmol/l)	39.0 (14.4)	35.9 (12.3)	43.0 (16.0)	0.081
1α,25(OH)_2_D_3_ (pg/ml)	46.9 (16.2)	49.0 (17.1)	44.0 (14.8)	0.297
GFR (ml/min/1.73 m^2^)	95.3 (20.6)	96.8 (22.1)	93.3 (18.9)	0.369
ASAT (U/l)	28.0 (13.8)	30.3 (16.9)	25.0 (7.6)	0.143
ALAT (U/l)	36.4 (20.8)	39.8 (24.7)	32.1 (13.9)	0.175
γ-GT (U/l)	41.3 (41.1)	30.5 (19.0)	55.1 (55.9)	*0.025*
Total protein (g/l)	70.1 (5.1)	70.5 (4.8)	69.6 (5.4)	0.545
Albumin (g/dl)	45.4 (5.0)	45.8 (4.9)	44.8 (5.1)	0.480
TC (mg/dl)	198.2 (46.9)	189.1 (25.0)	209.8 (63.9)	0.123
HDL (mg/dl)	47.3 (12.3)	50.2 (11.3)	43.5 (12.7)	*0.050*
TG (mg/dl)	155.8 (79.8)	129.9 (46.3)	188.8 (100.4)	*0.008*
WBC (cell/ml)	8.4 (2.1)	8.5 (2.2)	8.2 (1.9)	0.691
Platelets (cell/ml)	281.2 (64.1)	283.4 (69.9)	278.5 (57.6)	0.785
hsCRP (mg/dl)	0.9 (0.7)	0.8 (0.7)	1.0 (0.8)	0.486
Glucose (mg/dl)	108.7 (36.4)	94.4 (18.7)	127.0 (44.9)	*0.001*
Insulin (μU/l)	24.0 (11.9)	21.6 (10.7)	27.1 (12.9)	0.103
C-peptide (ng/ml)	4.0 (0.8–7.6)	3.8 (1.1)	4.2 (1.6)	0.285
HbA1c (rel.%)	6.0 (1.3)	5.4 (0.8)	6.7 (1.4)	*< 0.001*
HOMA2-IR	3.1 (1.6)	2.8 (1.4)	3.7 (1.8)	*0.050*
Significant fibrosis (*F* ≥ 2)	30 %	21 %	79 %	*0.001*
Advanced fibrosis (*F* ≥ 3)	13 %	17 %	83 %	*0.025*
Steatosis (*S* ≥ 1)	83 %	60 %	40 %	0.583
NASH	72 %	58 %	42 %	0.806

Data expressed as mean and standard deviations and percentages; *p* < 0.05 shown in italics

*BMI* body mass index, *WC* waist circumference, *Ca* calcium, *PTH* parathyroid hormone, *25*(*OH*)*D*_*3*_ 25-hydroxy vitamin D_3_, *1α*, *25*(*OH*)_*2*_ *D*_*3*_ 1α,25-dihydroxy vitamin D_3_, *GFR* glomerular filtration rate, *ASAT* aspartate aminotransferase, *ALAT* alanine aminotransferase, *γ-GT* γ-glutamyl transferase, *TC* total cholesterol, *HDL* high density lipoprotein, *TG* triglycerides, *WBC* white blood cells, *hsCRP* high sensitive c-reactive protein, *HbA1c* glycated hemoglobin, *HOMA2-IR* homeostatic model assessment insulin resistance, *NASH* non-alcoholic steatohepatitis

### Hepatic Histopathology

**Fig. 1:**
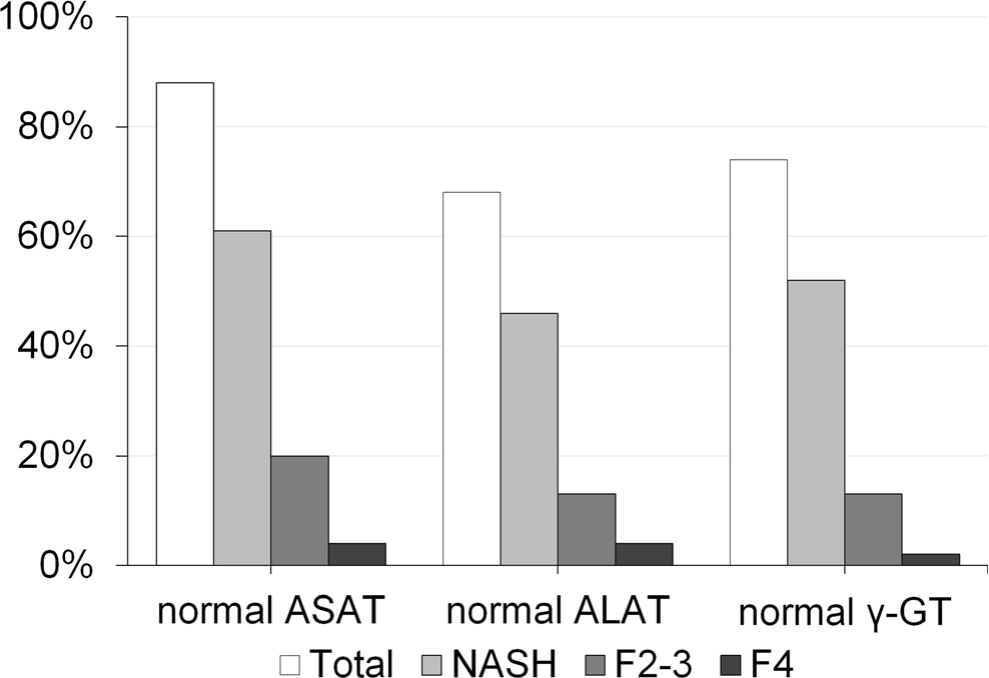
Patients with normal serum levels of liver enzymes divided by those with NASH, significant fibrosis (F2-3), and cirrhosis (F4). Note: *ASAT* aspartate aminotransferase, *ALAT* alanine aminotransferase, *γ-GT* γ-glutamyl transferase, *NASH* non-alcoholic steatohepatitis, *F2–F3* significant fibrosis, *F4* cirrhosis

Steatosis (≥5 %) was present in 83 %, lobular inflammation (≥1 foci/×200) in 80 %, and hepatocyte ballooning in 91 % of all biopsies. Diagnosis of NASH was found in 72 %, simple steatosis without NASH in 11 %, and normal liver morphology in 17 %. Significant fibrosis (*F* ≥ 2) was observed in 30 % of the study population with 9 % of advanced fibrosis (F3) and 4 % of cirrhosis (F4). Among patients with significant fibrosis (*F* ≥ 2), 2 % demonstrated simple steatosis without NASH and 28 % NASH. Moreover, all cirrhotic patients showed NASH. Figure 1 shows patients with normal serum levels of liver enzymes with NASH, F2–3, and cirrhosis (F4). Study participants with aspartate transaminase (ASAT) levels above the upper limit of normal (male >50 and female >35 U/l) demonstrated significantly higher glucose metabolism-related parameters compared to those with normal levels (*fasting glucose* 138.7 (39.6) vs. 104.7 (34.4) mg/dl, *p* = 0.005; *insulin* 33.0 (19.0) vs. 22.8 (10.4) μU/l, *p* = 0.048; *HbA1c* 6.9 (1.9) vs. 5.8 (1.1) rel.%, *p* = 0.050; *HOMA2-IR* 4.5 (2.6) vs. 3.0 (1.4), *p* = 0.033).

### Non-invasive Fibrosis Markers

**Fig. 2:**
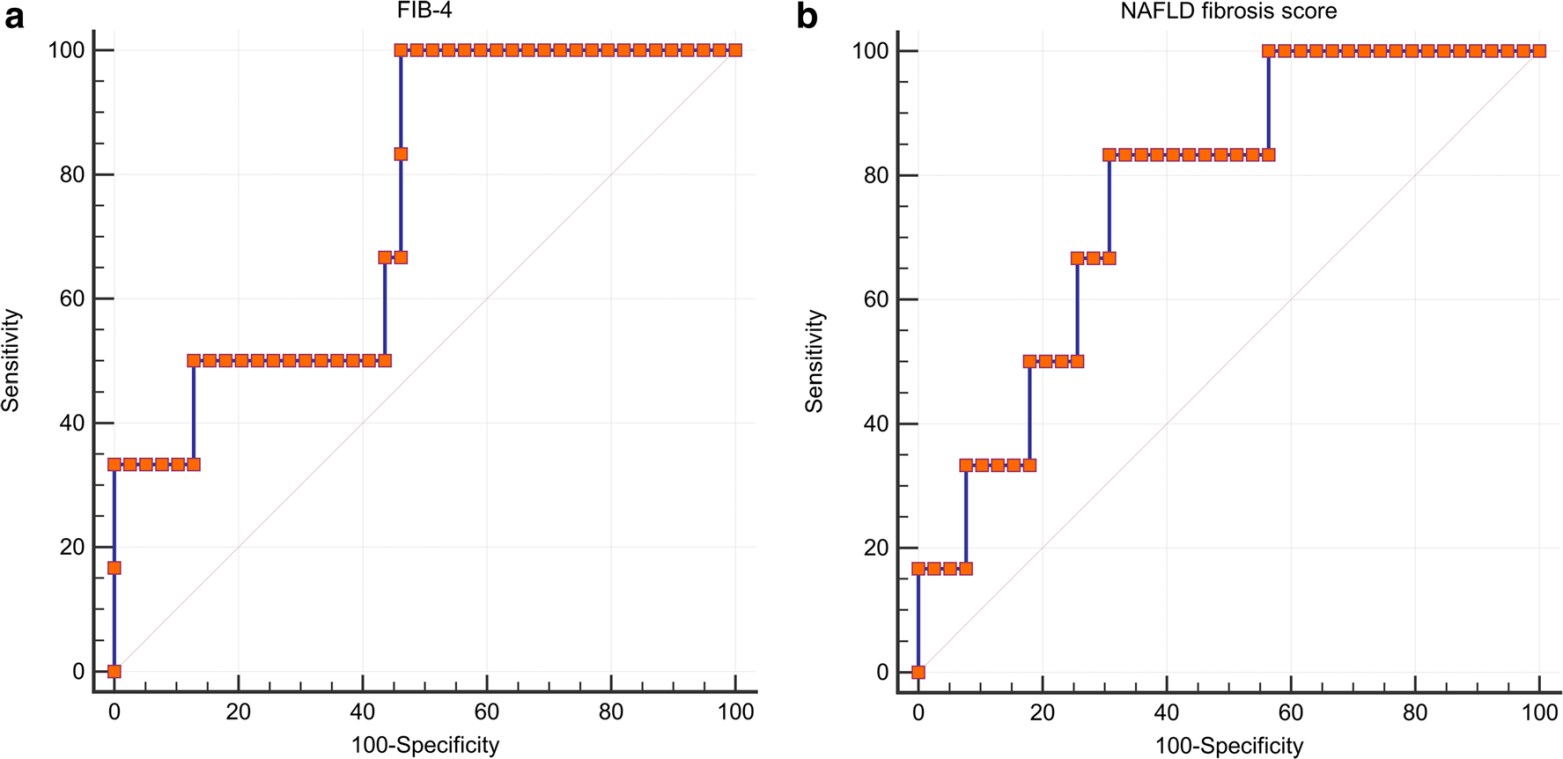
Receiver operating characteristic (ROC) curves of calculated non-invasive fibrosis markers FIB-4 index (**a**) and NAFLD fibrosis score (NFS) (**b**). Note: *NAFLD* non-alcoholic fatty liver disease

Calculated non-invasive fibrosis markers are able to clearly distinguish between *F* ≤ 2 and *F* ≥ 3 as significant differences in FIB-4 with 11.4 (standard error of 4.3) vs. 17.4 (8.0) (*p* = 0.007) and NFS with −29.1 (3.8) vs. −25.4 (3.3) (*p* = 0.028) could be observed. The ROC curves of FIB-4 and NFS (Fig. 2a, b) for predicting fibrosis stage *F* ≥ 3 demonstrated an AUC of 0.77 (0.09) and 0.75 (0.10). The FIB-4 <10.62 had a NPV of 100 % (sensitivity 100 %; specificity 54 %) and the NFS < −26.93 a NPV of 96 % (sensitivity 83 %; specificity 69 %).

### Vitamin D Metabolites

In our population, 76 % demonstrated vitamin D insufficiency (50–75 nmol/l), and 24 % deficiency (<50 nmol/l). By using multiple linear regression, accounting for 34 % of the variance (*R*^2^ = 0.335), 25(OH)D_3_ was associated with season (summer; *β* = 0.418, *p* = 0.002) and age (*β* = 0.341, *p* = 0.013). Moreover, 1α,25(OH)_2_D_3_ (*R*^2^ = 0.370) was associated with glomerular filtration rate (GFR; *β* = 0.523, *p* < 0.001) and, to a lesser extent, with waist circumference (WC; *β* = 0.289, *p* = 0.020).

### Risk Factors for NAFLD Severity and Fibrosis

**Fig. 3:**
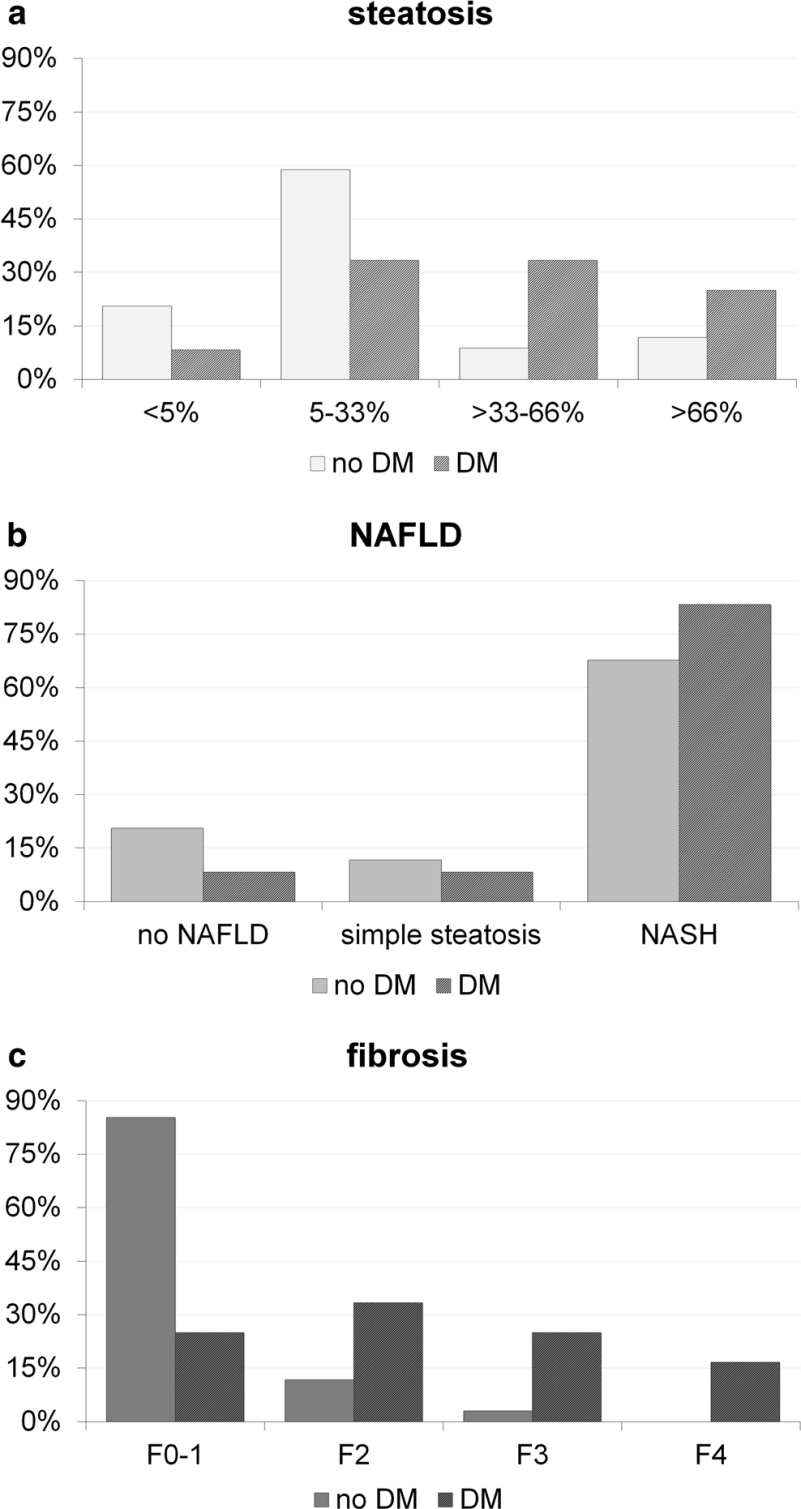
Prevalence of distinct histological forms of steatosis (**a**), non-alcoholic fatty liver disease (**b**), and fibrosis (**c**) according to the presence of diabetes mellitus in the study population. Note: *DM* type 2 diabetes mellitus, *NAFLD* non-alcoholic fatty liver disease, *NASH* non-alcoholic steatohepatitis

**Fig. 4:**
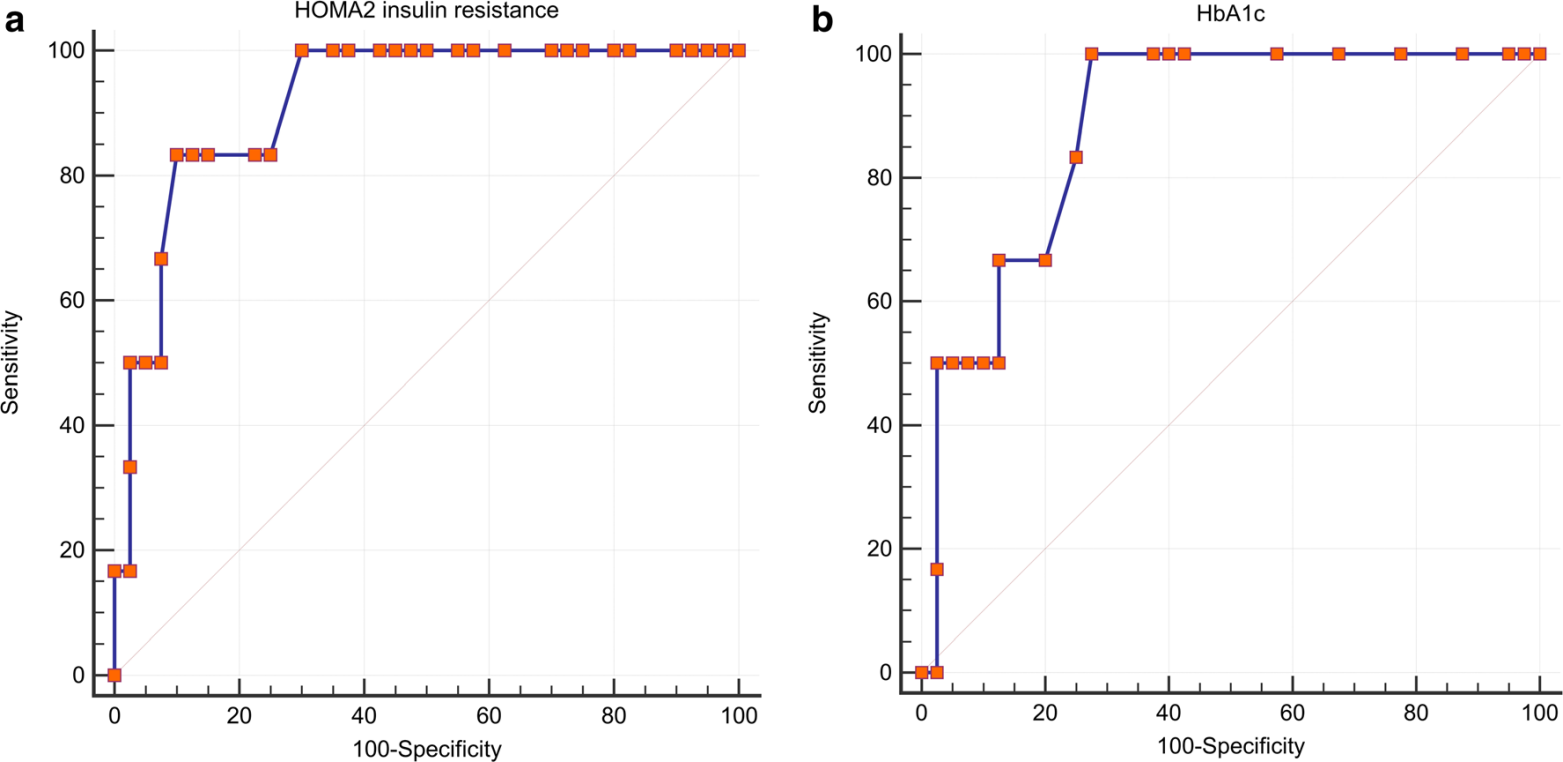
Receiver operating characteristic (ROC) curves of HOMA2-insulin resistance (**a**) and HbA1c (**b**). Note: *HOMA2-IR* homeostatic model assessment insulin resistance, *HbA1c* glycated hemoglobin

Figure 3 shows the prevalence of histological forms of steatosis, NAFLD, and fibrosis according to the presence of DM in the study population with significant differences in fibrosis. Notably, we found a statistically significant difference between non-diabetics and diabetics in the fibrosis stages (*p* = 0.001), but not in steatosis and NAFLD. Variables leading to higher levels of steatosis, fibrosis, and NAS score (adjusted for age and sex) in the study population are shown in Table 2. Steatosis and total NAS score was primarily associated with lower levels of mean corpuscular hemoglobin (MCH), higher alanine transaminase (ALAT) and WC. Moreover, fibrosis was primarily associated with higher levels of HOMA2-IR, procollagen type I propeptide (P1NP), lower osteocalcin, albumin-corrected calcium, PTH, male sex, lower 25(OH)D_3_ levels, and higher age. By using ROC curves, HOMA2-IR and HbA1c were able to significantly predict advanced fibrosis (*F* ≥ 3) with accuracy of 0.919 (0.048, *p* < 0.001 (Fig. 4a)) and 0.885 (0.056, *p* < 0.001 (Fig. 4b)). A HOMA2-IR >4.1 had a NPV of 97 % and a PPV of 55 % (sensitivity 83 %; specificity 90 %) and HbA1c >5.8 % of 35 % (sensitivity 100 %; specificity 73 %) but a PPV of 100 %. By using logistic regression analysis, other independent risk factors for advanced fibrosis were DM with an odds ratio (OR) of 12.8 (95 % confidence interval (CI), 1.2–137.4), *p* = 0.035 (adjusted for age) and MeS with an OR of 9.3 (0.99–87.5), *p* = 0.052.

**Table 2:** Multiple linear regression analyses of independent variables associated with fibrosis, steatosis, and NAS score

Model (backward selection)		Fibrosis *R* ^2^ = 0.805		Steatosis *R* ^2^ = 0.551		NAS score *R* ^2^ = 0.555	
		*ß*	*p*	*ß*	*p*	*ß*	*p*
Characteristics	Age (years)	0.206	*0.024*				
	Sex (male)	−0.286	*0.024*				
	WC (cm)			0.284	*0.016*	0.218	*0.055*
Vitamin D	25(OH)D_3_ (nmol/l)	−0.255	*0.006*				
Biochemical	Corr. Ca (mmol/L)	−0.298	*0.001*				
Bone turnover	P1NP (ng/ml)	0.401	*<0.001*				
	Osteocalcin (ng/ml)	−0.325	*0.005*				
	PTH (pg/ml)	−0.290	*0.004*				
Insulin resistance	HOMA2-IR	0.474	*<0.001*				
Liver	ALAT (U/l)			0.340	*0.007*	0.402	*0.001*
Blood count	MCH (pg)			−0.437	*<0.001*	−0.474	*<0.001*

*p* < 0.05 shown in italics

*WC* waist circumference, *25*(*OH*)*D*_*3*_ 25-hydroxy vitamin D_3_, *Ca* calcium, *P1NP* procollagen type I propeptides, *PTH* parathyroid hormone, *HOMA2-IR* homeostatic model assessment insulin resistance, *ALAT* alanine aminotransferase, *MCH* mean corpuscular hemoglobin, *ß* standardized beta coefficient, *p* value

## Discussion

In this prospective study, we observed a high prevalence of NASH (72 %), advanced fibrosis (13 %), and cirrhosis (4 %) within the reported range in bariatric patients of other studies despite a lower preoperative BMI, while the overall prevalence of steatosis (83 %) was slightly lower than previously estimated (85–98 %) [5]. Notably, the majority had normal levels liver transaminases with one quarter (27 and 23 %) in the upper tertile. Bariatric patients with MeS demonstrated ninefold higher odds for fibrosis and were older compared to those without MeS. Diabetic subjects had an even 13-fold higher odds ratio for advanced fibrosis and presented with higher fibrosis stages. Regarding vitamin D, a quarter suffered from deficiency (<50 nmol/l) and three quarters from insufficiency (50–75 nmol/l), which is in line with previously published data describing a 96 % prevalence of insufficiency/deficiency (<75 nmol/l) [23]. Overall, eight factors (higher HOMA2-IR, P1NP, age, male sex, lower osteocalcin, corrected Ca, PTH, and 25(OH)D_3_) were independently associated with biopsy-proven liver fibrosis in our study population.

As vitamin D is hydroxylated by the liver and afterwards converted into the active form in the kidney [24], 1α,25(OH)_2_D_3_ was primarily associated with higher levels of GFR in our population. Since the liver is involved in vitamin D metabolism, it may be assumed that patients with chronic liver disease show a high prevalence of vitamin D deficiency [6]. In our study, lower 25(OH)D_3_ levels were associated with higher stages of fibrosis, which is in line with previously published studies [9, 25, 26]. As serum vitamin D correlates with a fibrogenic state in the liver, vitamin D may be an antifibrotic treatment option in liver fibrosis [25]. Accordingly, special attention should be paid to vitamin D deficiency particularly regarding fibrosis progression, which can provoke chronic inflammation and subsequent induction of liver cell apoptosis [27] and may have synergistic effects with insulin resistance [7]. Moreover, the bone turnover marker P1NP might be useful in predicting or monitoring liver fibrosis, respectively, as during fibrogenesis type I collagen levels increase up to eightfold [28, 29]. Moreover, PTH and stages of fibrosis showed a negative association. In our study, all patients suffered from VDD but in only 4 patients (8 %) PTH level was increased. The cause of normal to low PTH levels in patients with VDD and liver disease is unclear [30]. An explanation could be specific vitamin D-receptor gene polymorphisms which might cause suppression of PTH secretion [30, 31].

There is rising evidence that patients with advanced and often undetected fibrosis demonstrate increased rates of mortality [32] necessitating the identification of significant indicators of fibrosis in morbidly obese patients. Indeed, this analysis demonstrates that patients with impaired glucose metabolism parameters along with vitamin D/bone turnover markers and/or DM or MeS, respectively, might be considered as high risk patients. However, these indicators in this relatively small study population require prospective validation by further larger clinical studies. Performing a liver biopsy in all bariatric patients might be practically not feasible and too invasive. Therefore, non-invasive fibrosis markers with a good accuracy predicting advanced fibrosis could be useful to identify patients at higher risk preoperatively [19, 20]. Including additional metabolic indicators or perhaps even transient elastography [33] might help identifying these patients. In clinical practice, we suggest to identify these risk factors preoperatively and discuss the potential implications for the planned surgical procedure in a multidisciplinary team involving surgeons, internists (endocrinologists, hepatologists), and nutritionists. If a patient is classified as high risk, further investigation with transient elastography eventually followed by liver biopsy appears justified. With this approach, it is not necessary to perform liver biopsy in all bariatric patients. It may be useful to develop an algorithm for investigation of patients at risk and the potential consequences for modification of postponing the surgical procedure. Nevertheless, this needs to be precisely assessed in a prospective study.

Emerging evidence suggests that rapid weight loss during bariatric surgery with mobilization of potentially lipotoxic fatty acids might adversely affect the liver [34]. Therefore, patients with advanced fibrosis/cirrhosis may be at increased peri- and postoperative risk for hepatic dysfunction [35]. Another explanation might be a lack of sufficient supplementation of macro- and micronutrients [34], e.g., vitamin D.

The strengths of this study are the biopsy-proven liver disease, the well-characterized study sample, and the comprehensive laboratory data. Among the limitations are the relatively small sample size and thus limited applicability to patients with body weight below 140 kg and vitamin D deficiency. This population, however, might represent a significant proportion of patients undergoing bariatric surgery. Moreover, due to the inclusion criteria of VDD (<75 nmol/l) in this randomized controlled trial [11], no comparison between patients with normal vitamin D levels and those with VDD was possible.

## Conclusions

The present study shows that significant fibrosis is frequent in vitamin D deficient, morbidly obese patients with concurrent DM, and/or MeS. Additionally, increased levels of IR, P1NP, lower osteocalcin, and vitamin D deficiency are associated with increased stages of fibrosis. Based on our results, we suggest that patients with preoperative presence of such markers and high scores of non-invasive fibrosis markers are at high risk for liver fibrosis. As a consequence, rigorous monitoring regarding liver function and avoidance of macro- and micronutrient deficiencies, in particular calcium, vitamin D, and protein, by sufficient nutritional counseling must be guaranteed in a multidisciplinary team.

## References

[1] Masuoka HC, Chalasani N (2013). Nonalcoholic fatty liver disease: an emerging threat to obese and diabetic individuals. Ann N Y Acad Sci.

[2] Brunt EM, Tiniakos DG (2010). Histopathology of nonalcoholic fatty liver disease. World J Gastroenterol.

[3] Fabbrini E, Sullivan S, Klein S (2010). Obesity and nonalcoholic fatty liver disease: biochemical, metabolic, and clinical implications. Hepatology.

[4] de Marco R, Locatelli F, Zoppini G, Verlato G, Bonora E, Muggeo M (1999). Cause-specific mortality in type 2 diabetes. The Verona Diabetes Study. Diabetes Care.

[5] Machado M, Marques-Vidal P, Cortez-Pinto H (2006). Hepatic histology in obese patients undergoing bariatric surgery. J Hepatol.

[6] Putz-Bankuti C, Pilz S, Stojakovic T, Scharnagl H, Pieber TR, Trauner M (2012). Association of 25-hydroxyvitamin D levels with liver dysfunction and mortality in chronic liver disease. Liver Int.

[7] Eliades M, Spyrou E, Agrawal N, Lazo M, Brancati FL, Potter JJ (2013). Meta-analysis: vitamin D and non-alcoholic fatty liver disease. Aliment Pharmacol Ther.

[8] Eliades M, Spyrou E (2015). Vitamin D: a new player in non-alcoholic fatty liver disease?. World J Gastroenterol.

[9] Grunhage F, Hochrath K, Krawczyk M, Hoblinger A, Obermayer-Pietsch B, Geisel J (2012). Common genetic variation in vitamin D metabolism is associated with liver stiffness. Hepatology.

[10] Vander Naalt SJ, Gurria JP, Holterman AL (2014). Surgical treatment of nonalcoholic fatty liver disease in severely obese patients. Hepat Med.

[11] Luger M, Kruschitz R, Marculescu R, Haslacher H, Hoppichler F, Kallay E (2015). The link between obesity and vitamin D in bariatric patients with omega-loop gastric bypass surgery—a vitamin D supplementation trial to compare the efficacy of postoperative cholecalciferol loading (LOAD): study protocol for a randomized controlled trial. Trials.

[12] Dale O, Salo M (1996). The Helsinki Declaration, research guidelines and regulations: present and future editorial aspects. Acta Anaesthesiol Scand.

[13] Schulz KF, Altman DG, Moher D, Group C (2010). CONSORT 2010 Statement: updated guidelines for reporting parallel group randomised trials. Trials.

[14] Jain A, Bhayana S, Vlasschaert M, House A (2008). A formula to predict corrected calcium in haemodialysis patients. Nephrol Dial Transplant.

[15] Veith R (2006). What is the optimal vitamin D status for health?. Prog Biophys Mol Biol.

[16] Klahr S, Levey AS, Beck GJ, Caggiula AW, Hunsicker L, Kusek JW (1994). The effects of dietary protein restriction and blood-pressure control on the progression of chronic renal disease. Modification of Diet in Renal Disease Study Group. N Engl J Med.

[17] Levy JC, Matthews DR, Hermans MP (1998). Correct homeostasis model assessment (HOMA) evaluation uses the computer program. Diabetes Care.

[18] International Diabetes Federation. The IDF consensus worldwide definition of the metabolic syndrome. IDF. 2006.

[19] McPherson S, Stewart SF, Henderson E, Burt AD, Day CP (2010). Simple non-invasive fibrosis scoring systems can reliably exclude advanced fibrosis in patients with non-alcoholic fatty liver disease. Gut.

[20] Angulo P, Hui JM, Marchesini G, Bugianesi E, George J, Farrell GC (2007). The NAFLD fibrosis score: a noninvasive system that identifies liver fibrosis in patients with NAFLD. Hepatology.

[21] Kleiner DE, Brunt EM, Van Natta M, Behling C, Contos MJ, Cummings OW (2005). Nonalcoholic Steatohepatitis Clinical Research, N. Design and validation of a histological scoring system for nonalcoholic fatty liver disease. Hepatology.

[22] Bedossa P, Poitou C, Veyrie N, Bouillot JL, Basdevant A, Paradis V (2012). Histopathological algorithm and scoring system for evaluation of liver lesions in morbidly obese patients. Hepatology.

[23] Luger M, Kruschitz R, Langer F, Prager G, Walker M, Marculescu R (2015). Effects of omega-loop gastric bypass on vitamin D and bone metabolism in morbidly obese bariatric patients. Obes Surg.

[24] Tsuneoka K, Tameda Y, Takase K, Nakano T (1996). Osteodystrophy in patients with chronic hepatitis and liver cirrhosis. J Gastroenterol.

[25] Beilfuss A, Sowa JP, Sydor S, Beste M, Bechmann LP, Schlattjan M (2015). Vitamin D counteracts fibrogenic TGF-beta signalling in human hepatic stellate cells both receptor-dependently and independently. Gut.

[26] Targher G, Bertolini L, Scala L, Cigolini M, Zenari L, Falezza G (2007). Associations between serum 25-hydroxyvitamin D3 concentrations and liver histology in patients with non-alcoholic fatty liver disease. Nutr Metab Cardiovasc Dis.

[27] Zhu L, Kong M, Han YP, Bai L, Zhang X, Chen Y (2015). Spontaneous liver fibrosis induced by long term dietary vitamin D deficiency in adult mice is related to chronic inflammation and enhanced apoptosis. Can J Physiol Pharmacol.

[28] Guanabens N, Pares A, Alvarez L, Martinez de Osaba MJ, Monegal A, Peris P (1998). Collagen-related markers of bone turnover reflect the severity of liver fibrosis in patients with primary biliary cirrhosis. J Bone Miner Res.

[29] Veidal SS, Vassiliadis E, Bay-Jensen AC, Tougas G, Vainer B, Karsdal MA (2010). Procollagen type I N-terminal propeptide (PINP) is a marker for fibrogenesis in bile duct ligation-induced fibrosis in rats. Fibrogenesis Tissue Repair.

[30] Miroliaee A, Nasiri-Toosi M, Khalilzadeh O, Esteghamati A, Abdollahi A, Mazloumi M (2010). Disturbances of parathyroid hormone-vitamin D axis in non-cholestatic chronic liver disease: a cross-sectional study. Hepatol Int.

[31] Adorini L (2009). Vitamin D receptor polymorphisms in primary biliary cirrhosis: a functional connection?. J Hepatol.

[32] Kim D, Kim WR, Kim HJ, Therneau TM (2013). Association between noninvasive fibrosis markers and mortality among adults with nonalcoholic fatty liver disease in the United States. Hepatology.

[33] Petta S, Vanni E, Bugianesi E, Di Marco V, Camma C, Cabibi D (2015). The combination of liver stiffness measurement and NAFLD fibrosis score improves the noninvasive diagnostic accuracy for severe liver fibrosis in patients with nonalcoholic fatty liver disease. Liver Int.

[34] Hafeez S, Ahmed MH (2013). Bariatric surgery as potential treatment for nonalcoholic fatty liver disease: a future treatment by choice or by chance?. J Obes.

[35] Bhangui P, Laurent A, Amathieu R, Azoulay D (2012). Assessment of risk for non-hepatic surgery in cirrhotic patients. J Hepatol.

